# Acute Kidney Injury From Biopsy-Proven Renal Oxalosis From Excessive Intake of Vitamin C Leading to End-Stage Kidney Disease

**DOI:** 10.7759/cureus.33061

**Published:** 2022-12-28

**Authors:** Ryan Williams, Mujahed Dauleh, Catherine Abendroth, Gurwant Kaur

**Affiliations:** 1 Medicine, Penn State Health Milton S. Hershey Medical Center, Hershey, USA; 2 Pathology, Penn State Health Milton S. Hershey Medical Center, Hershey, USA; 3 Medicine/Nephrology, Penn State Health Milton S. Hershey Medical Center, Hershey, USA

**Keywords:** acute kidney injury, vitamin c toxicity, end stage kidney disease, hyperoxaluria, oxalosis

## Abstract

We are presenting a case of a 55-year-old Caucasian female who presented with acute kidney injury requiring hemodialysis. Her native kidney biopsy showed extensive crystals in both the cortex and medulla, morphologically consistent with calcium oxalate crystals. The etiology was attributed to vitamin C-induced kidney hyperoxaluria. She has remained hemodialysis dependent for more than three months since the initial presentation, establishing a diagnosis of end-stage kidney disease.

## Introduction

We report a case of a 55-year-old woman who presented with acute kidney injury (AKI) with life-threatening electrolyte abnormalities and was found to have renal oxalosis on a native kidney biopsy. She has remained dialysis-dependent and has developed end-stage kidney disease (ESKD). The etiology of unresolved AKI leading to ESKD was attributed to vitamin C-induced kidney hyperoxaluria. The continued dependence on dialysis sets this case apart from the majority of the documented cases of hyperoxaluria following vitamin C intake [1,2].

This article was previously presented as a poster at the 2021 American Society of Nephrology (ASN) meeting on November 4, 2021.

## Case presentation

A 55-year-old woman with a past medical history including chronic kidney disease (with an available baseline serum creatinine of 2 mg/dL four months prior and 0.73 mg/dL four years before this presentation) and hypothyroidism complicated by prior myxedema coma not compliant with medications presented after a post-syncopal fall. Initial vitals were notable for a temperature of 33.9 ℃ with bradycardia of 30 beats per minute. Physical examination was notable for generalized lethargy, confusion, and ecchymosis over the left mandible with accompanying swelling of the lips and the chin. Initial labs are shown in Table 1. Her presenting serum creatinine was 35.30 mg/dl along with serum potassium of 7 mmol/L. Her urine output was minimal and she soon became anuric. 

**Table 1:** Laboratory findings BUN: blood urea nitrogen, pCO2: partial pressure of carbon dioxide, pO2: partial pressure of oxygen

Labs	Day of admission (day 0)	After 3 sessions of Hemodialysis	Recent (1 year after initial presentation, on dialysis)	Normal range
Sodium	124	133	138	136 – 145 mmol/L
Potassium	7	3.8	4.9	3.5 – 5.1 mmol/L
Bicarbonate	8	23	24	22 – 29 mmol/L
Chloride	81	91	97	98 – 107mmol/L
Anion Gap	41	19	17	5 – 14
BUN	194	90	49	6 – 23 mg/dL
Creatinine	35.30	17.05	6.76	0.6 – 1 mg/dL
Phosphorus	15.4	7.7	5.2	2.5 – 4.5 mmol/L
Calcium	9	7.9	10.4	8.4 – 10.2 mg/dL
pH	7.192			7.35 – 7.45
pCO2	25			35 – 45 mmHg
pO2	34			80 – 105 mmHg
Lactic	2.3			0.5 – 2.2 mmol/L
Cortisol	23.5			2.3 – 19.5 ug/dL
Thyroid-stimulating hormone	>100.0			0.3 – 4.2 uIU/mL
Free T4	<0.1	0.87		0.9 – 1.7 ng/dL
Free T3	<0.5	1.4		2 – 4.4 ng/dL
Creatine phosphokinase	1798			26 – 192 unit/L
Prothrombin Time	14.1			12 – 14.2 seconds
International normalized ratio	1.1			0.9 – 1.1
C3	78			90 – 180 mg/dL
C4	22			10 – 40 mg/dL
Anti-neutrophil Antibody	<1:80			<1:80
Anti-neutrophil cytoplasmic antibody	Negative			Negative
Hepatitis B surface Antibody	Nonreactive			Nonreactive
Hepatitis B surface Antigen	Nonreactive			Nonreactive
Hepatitis B core antibody	Nonreactive			Nonreactive
Hepatitis C antibody	Nonreactive			Nonreactive
Gliadin Antibody IgG	1 Units			< 19 considered negative
Gliadin Antibody IgA	2 Units			< 19 considered negative
Tissue Transglutaminase IgA Antibody	<2 U/mL			< 3 considered negative
Anti Proteinase Antibody	2 AU/mL			< 19 considered negative

She was transferred to the intensive care unit (ICU) and was started on a bicarbonate drip for significant metabolic acidosis. She was also given Kayexalate. She received hydrocortisone and levothyroxine for treatment of presumed myxedema coma. Due to persistent anuria, acidosis, and worsening kidney function she was started on hemodialysis on the day of admission via a temporary dialysis catheter. 

Workup for her worsening kidney function included negative antinuclear antibody, antineutrophil cytoplasmic antibody, hepatitis B surface antigen, hepatitis B surface antibody, hepatitis B core antibody, hepatitis C antibody, human immunodeficiency virus antibody, and normal C3/C4 levels. 

**Figure 1:**
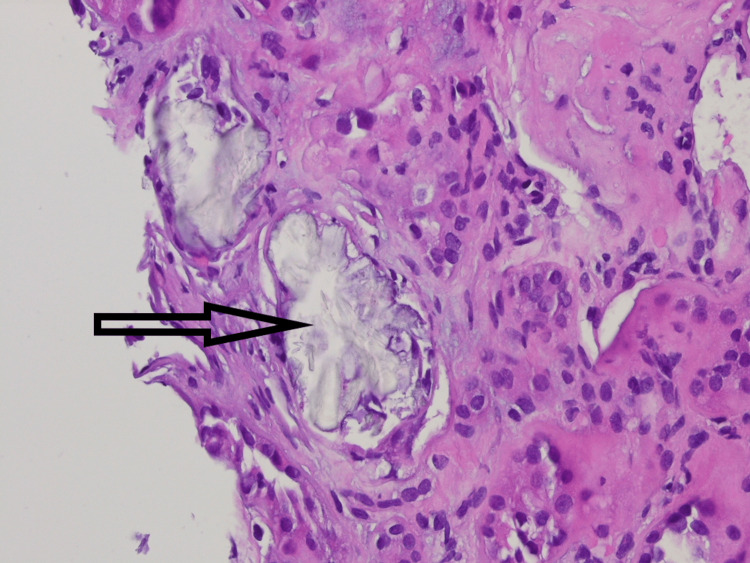
Kidney biopsy showing rhomboid-shaped calcium oxalate crystals (black arrow) distending renal tubule with attenuation and disruption of the epithelial lining (H&E x400)

**Figure 2:**
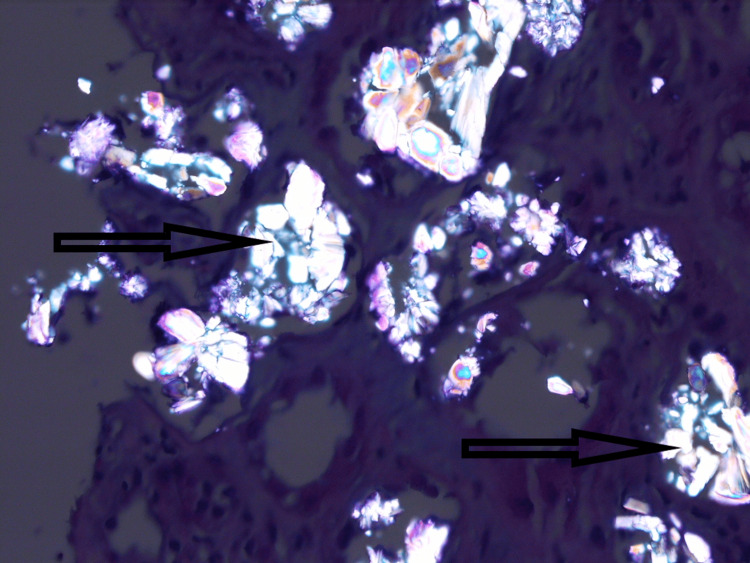
Kidney biopsy showing calcium oxalate crystals (black arrow) characteristically birefringent under polarized light microscopy (H&E, x250)

Her kidney ultrasound showed numerous small non-obstructing calculi bilaterally. A native kidney biopsy showed crystals in both the cortex and medulla with widespread interstitial fibrosis affecting more than 50% of the cortical area. A large number of tubules were found to be distended by rhomboid-shaped crystalline deposits (Figure 1). The crystals were birefringent under polarized light and stained negative with Von Kossa stain (Figure 2). These crystals were morphologically consistent with calcium oxalate crystals. Immunofluorescence and electron microscopy were non-contributory. 

Additional workup, directed to the etiology of her kidney oxalosis, consisted of ruling out fat malabsorption, ethylene glycol ingestion, thiamine deficiency, and chronic ingestion of oxalate precursor. Assessment of her fecal fat, tissue transglutaminase immunoglobulin (Ig) A antibody, anti-gliadin IgG, and anti-gliadin IgA were negative. The toxicology report showed no evidence of ethylene glycol ingestion. Serum osmolality wasn't sent at the time of admission and the osmolar gap could not be calculated. Her thiamine level was 62 nmol/L (normal 70-180 nmol/L). However, this mild level of depression would not be consistent with her degree of kidney oxalosis. Her outpatient medications included ascorbic acid 1000 mg daily, calcium 500 mg, digestive enzymes, docusate 100 mg, cranberry 500 mg capsule, a multivitamin (unknown composition), omeprazole 20 mg, Miralax 17 g daily, and vitamin D 5000 units daily. Given the largely negative workup, it is presumed that her kidney failure due to renal oxalosis was due to vitamin C toxicity. Serum oxalate levels were not obtained initially. The pathology report of her kidney biopsy came after she had received a few sessions of hemodialysis, and we assume that her levels would have improved after hemodialysis even if they were high initially. However, it is impossible to comment without the oxalate levels.

Her urine output began to improve, but she continued to require dialysis for electrolyte management. Her clinical status gradually improved and she was ultimately discharged. Attempts were made throughout the hospitalization to obtain a 24-hour urine collection for a metabolic stone panel. However, this wasn’t feasible due to low urine output. She continues to require hemodialysis greater than one year following discharge and is actively followed by the nephrology team in an outpatient dialysis unit. 

## Discussion

This case demonstrates an example of excess vitamin C intake resulting in hyperoxaluria leading to AKI and ESKD requiring dialysis. The diagnosis of hyperoxaluria often requires high clinical suspicion and often can be missed. In this case, the clinical presentation was complex given her initial presentation with myxedema coma with significant electrolyte abnormalities. She was promptly started on hemodialysis due to her oligo-anuric state and electrolyte abnormalities.

Hyperoxaluria is a condition characterized by increased urinary excretion of oxalate. Primary hyperoxaluria occurs when an inborn error in metabolism results in low enzymatic activity of specific enzymes involved in oxalate metabolism. The three subtypes of primary hyperoxaluria are categorized based on the affected enzyme. A defect in alanine-glyoxylate aminotransferase characterizes subtype 1, while subtypes 2 and 3 are characterized by the low enzymatic activity of glyoxylate reductase-hydroxypyruvate reductase and mitochondrial 4-hydroxy-2-oxoglutarate aldolase respectively [3]. Secondary hyperoxaluria occurs with increased dietary ingestion of oxalate or its precursors. In either case, when the accumulation of calcium oxalate exceeds the renal clearance, systemic deposition of calcium oxalate occurs. The effects of deposition vary based on the organ affected, but commonly affected organs include the kidneys, heart, bones, and vasculature.  

Secondary hyperoxaluria can occur from direct ingestion of excess oxalate, direct ingestion of oxalate precursors, or increased intestinal absorption of an otherwise normal dietary intake [4,5]. Previous literature has demonstrated that dietary intake of oxalate contributes to approximately 50% of the oxalate that is excreted in the urine, with the other 50% coming from endogenous synthesis [6]. Commonly implicated food sources contributing to excess oxalate intake include rhubarb, chocolate, and spinach, where an intake greater than 1000 mg/day has been shown to lead to an increased risk of developing hyperoxalosis [5]. Additional sources implicated in the development of hyperoxalosis include foods high in vitamin C and the ingestion of ethylene glycol. Vitamin C is a precursor of oxalate and excess levels can lead to subsequent hyperoxalosis, while ethylene glycol produces oxalate as a product of its breakdown [7]. Outside of the specific intakes, individuals affected by conditions leading to fat malabsorption are at increased risk of hyperoxaluria due to the formation of fatty acid complexes in the intestinal tract that facilitate the absorption of oxalate. The net effect in these patients is an increase in oxalate absorption from the gut. In these cases, excess oxalate intake is not necessarily needed to develop high systemic levels of oxalate. The presence of fat malabsorption alone can lead to increased absorption of oxalate, leading to systemic deposition.  

There were several contributing factors present at the time of her initial presentation that left her predisposed to complications. Metabolic acidosis lowers citrate clearance and leads to decreased levels of citrate within the tubular lumen [8]. As the primary inhibitor of calcium oxalate precipitation, diminished levels of citrate can lead to an increased incidence of oxalate precipitation. Although not profoundly acidotic at the time of presentation, her acidemia likely left her at increased risk of oxalate deposition in the tubules.  

Another important factor in working up the cases of vitamin C-induced hyperoxaluria is overall vitamin C intake. It is unclear from this case how much vitamin C she was taking before she arrived at the hospital or for how long she felt unwell before her syncopal event. Her medication history suggested that she was taking at least 1000 mg of vitamin C daily in the form of a vitamin C supplement and a multivitamin. However, given her history of medication non-compliance and intermittently taking her Synthroid, assessing her true intake before arrival was challenging. A clear understanding of the amount of vitamin C intake required to develop hyperoxaluria has not been identified. Prior case reports have shown the vitamin C intake required to cause hyperoxaluria varies, but has been as low as 680 mg daily [1]. A vitamin C dose of 1000 mg/d can lead to increased oxalate excretion by 6-13 mg/day [9].

The treatment efforts in patients suspected of having hyperoxaluria secondary to vitamin C toxicity should largely focus on limiting the intake of vitamin C. As mentioned previously, vitamin C is a precursor to oxalate, and limiting the overall intake of vitamin C is essential in preventing hyperoxaluria. While specific vitamin C supplements are relatively easy to avoid, there are many additional sources of vitamin C such as multivitamins, juices, fruits, peppers, etc. that are often overlooked. Referral to a dietician can help identify overlooked sources of vitamin C in the diet and can be beneficial in preventing recurrence. 

## Conclusions

It is important to consider the potential complications of vitamin C toxicity in patients, especially those with underlying chronic kidney disease. A retrospective review of her labs before her presentation suggests that she may have had undiagnosed underlying chronic kidney disease with a baseline creatinine of around 2 mg/dL. The complications from overexposure to vitamin C can have lifelong consequences in those with underlying kidney dysfunction.  

## References

[1] Rathi S, Kern W, Lau K (2007). Vitamin C-induced hyperoxaluria causing reversible tubulointerstitial nephritis and chronic renal failure: a case report. J Med Case Rep.

[2] Lamarche J, Nair R, Peguero A, Courville C (2011). Vitamin C-induced oxalate nephropathy. Int J Nephrol.

[3] Spasovski G, Beck BB, Blau N, Hoppe B, Tasic V (2010). Late diagnosis of primary hyperoxaluria after failed kidney transplantation. Int Urol Nephrol.

[4] Worcester EM (2002). Stones from bowel disease. Endocrinol Metab Clin North Am.

[5] Holmes RP, Kennedy M (2000). Estimation of the oxalate content of foods and daily oxalate intake. Kidney Int.

[6] Holmes RP, Goodman HO, Assimos DG (2001). Contribution of dietary oxalate to urinary oxalate excretion. Kidney Int.

[7] Stapenhorst L, Hesse A, Hoppe B (2008). Hyperoxaluria after ethylene glycol poisoning. Pediatr Nephrol.

[8] Simpson DP (1983). Citrate excretion: a window on renal metabolism. Am J Physiol.

[9] Daudon M, Jungers P (2004). Drug-induced renal calculi: epidemiology, prevention and management. Drugs.

